# Supplementary material for: Use of an In Vitro Skin Parallel Artificial Membrane Assay (Skin-PAMPA) as a Screening Tool to Compare Transdermal Permeability of Model Compound 4-Phenylethyl-Resorcinol Dissolved in Different Solvents
Source: Pharmaceutics. 2021 Oct 21;13(11):1758. doi: 10.3390/pharmaceutics13111758 (PMC8625559; doi:10.3390/pharmaceutics13111758)
Supplement: Supplementary file 1 [file pharmaceutics-13-01758-s001.zip › pharmaceutics-1391803-supplementary.pdf]

pharmaceutics

Supplementary Materials: Use of an In Vitro Skin Parallel Artificial Membrane Assay (Skin-PAMPA) As a Screening Tool to Compare Transdermal Permeability of Model Compound 4-Phenylethyl-Resorcinol Dissolved in Different Solvents

Bálint Sinkó, Vivien Bárdos, Dániel Vesztergombi, Szabina Kádár, Petra Malcsiner, Anne Moustie, Chantal Jouy, Krisztina Takács-Novák and Sebastien Grégoire

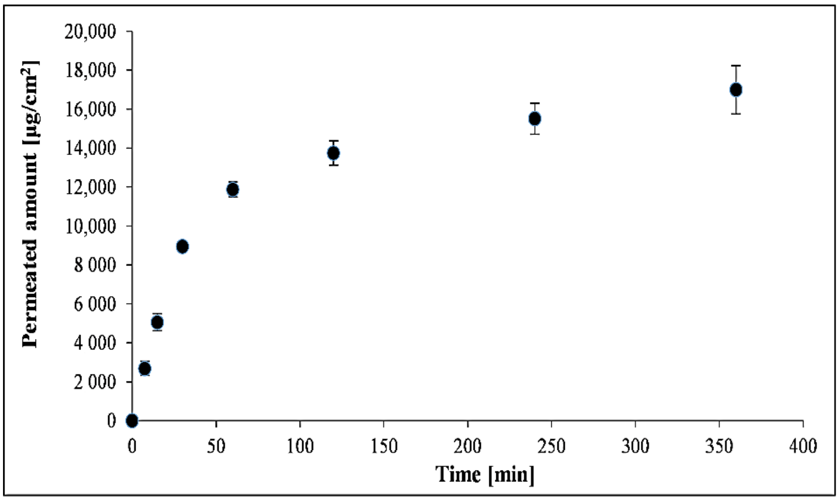

Figure S1. The permeability profile of PER dissolved in ethanol using Skin-PAMPA.

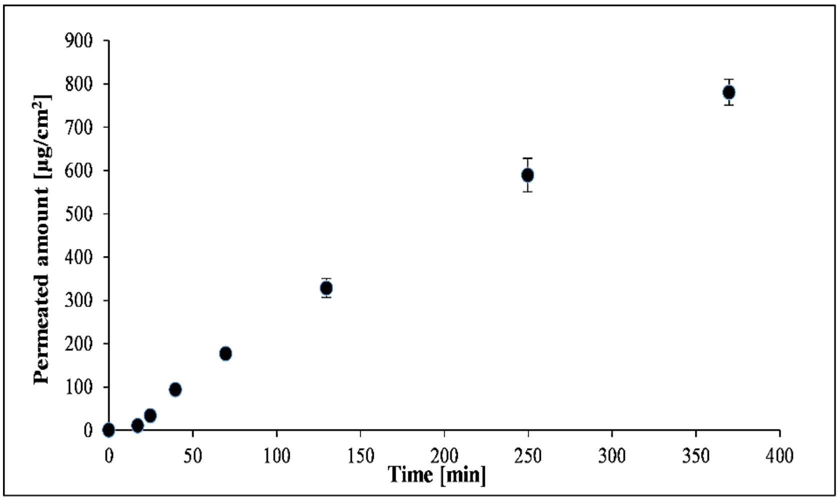

Figure S2. The permeability profile of PER dissolved in glycerol using Skin-PAMPA.

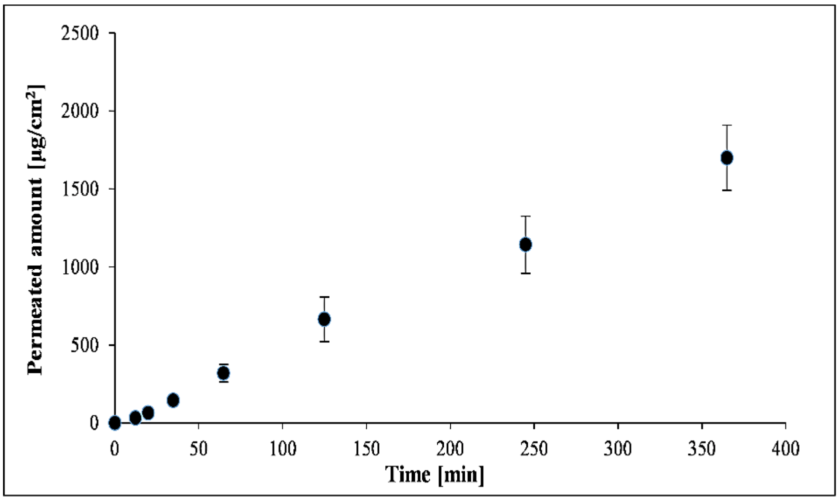

Figure S3. The permeability profile of PER dissolved in dimethylisosorbide using Skin-PAMPA.

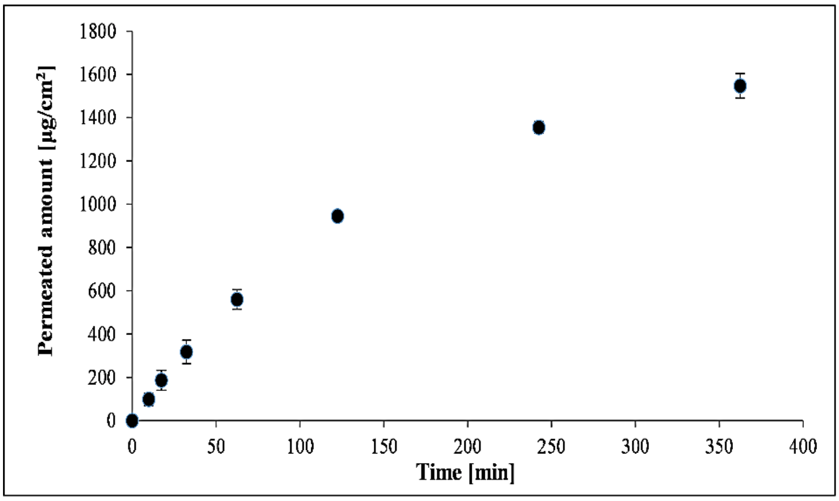

Figure S4. The permeability profile of PER dissolved in water/ethanol – 80:20 (*w/w*) using Skin-PAMPA.

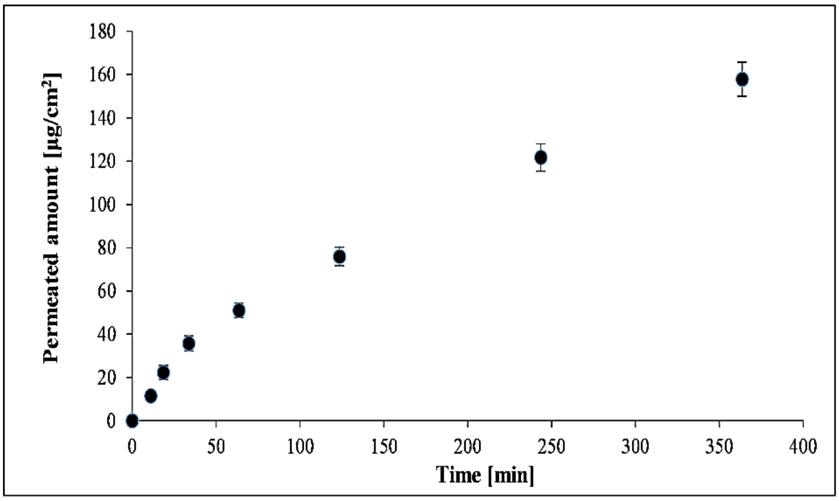

Figure S5. The permeability profile of PER dissolved in water/dimethylisosorbide – 90:10 (*w/w*) using Skin-PAMPA.

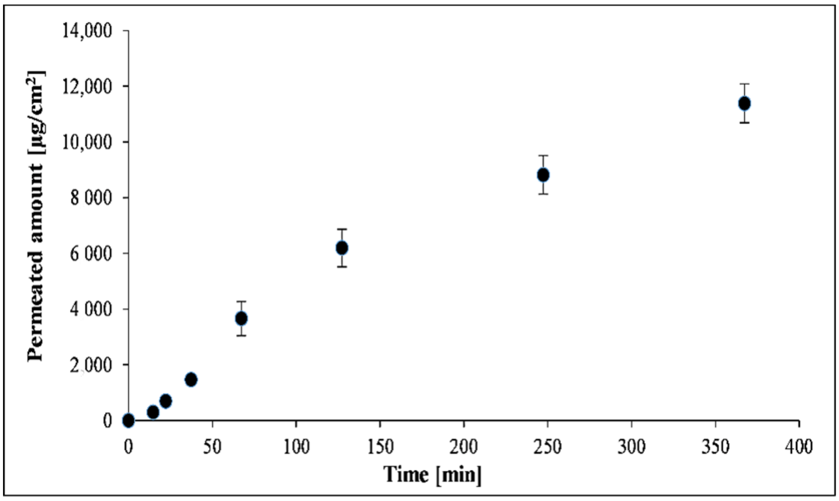

Figure S6. The permeability profile of PER dissolved in propylene glycol using Skin-PAMPA.

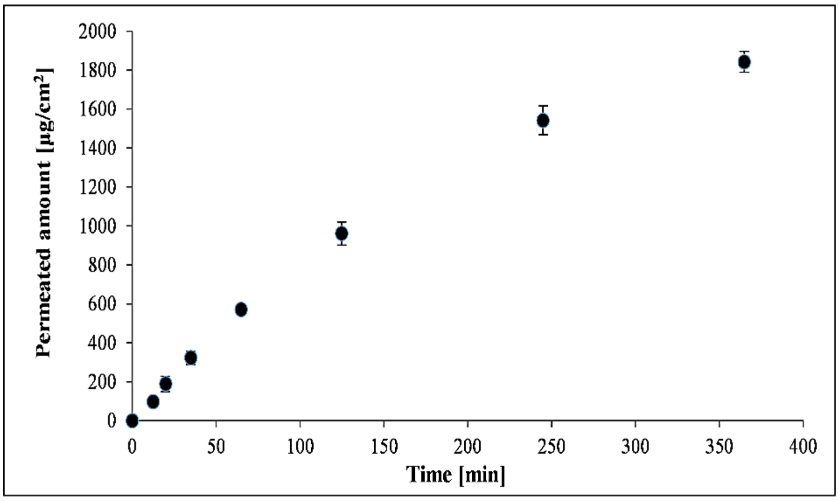

Figure S7. The permeability profile of PER dissolved in water:propylene glycol – 80:20 (*w/w*) using Skin-PAMPA.

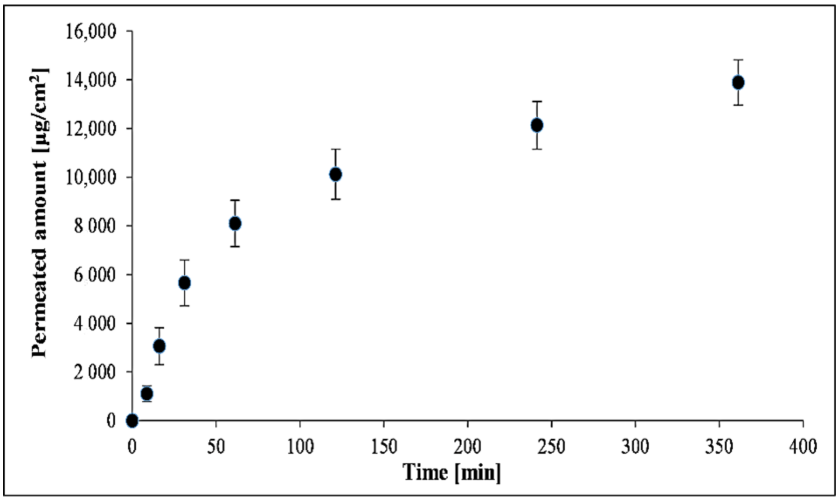

Figure S8. The permeability profile of PER dissolved in water/propylenglycol/ethanol – 10:30:60 (*w/w/w*) using Skin-PAMPA.

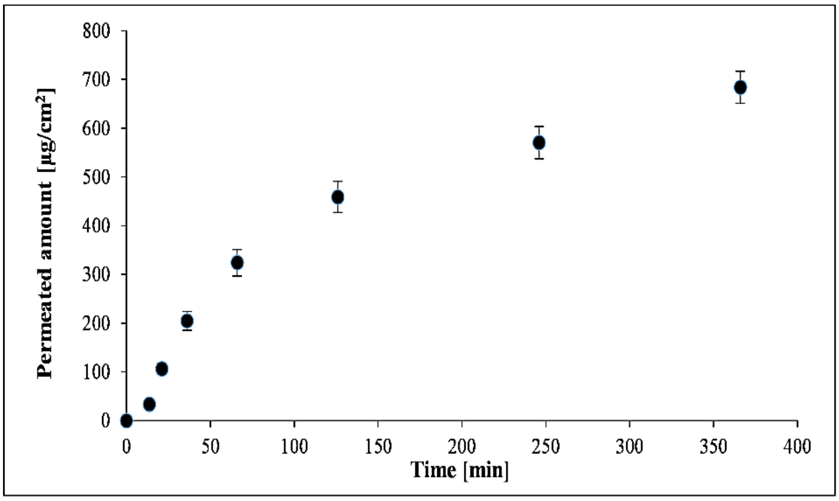

Figure S9. The permeability profile of PER dissolved in capric/caprylic triglycerides using Skin-PAMPA.

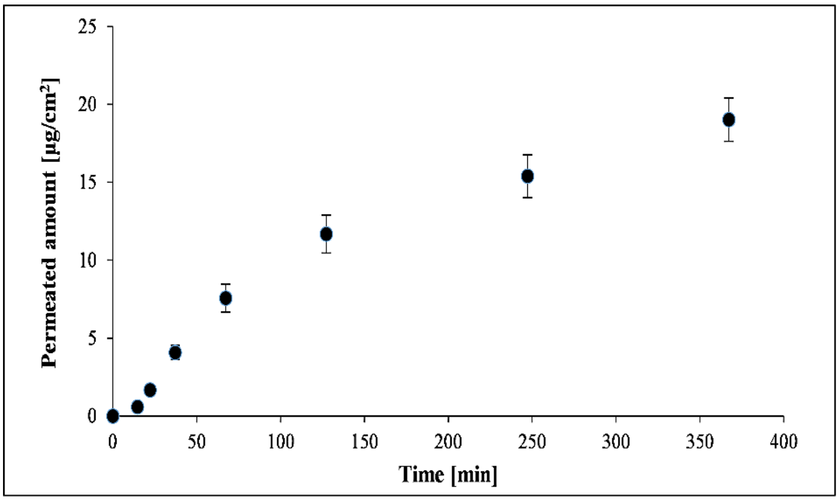

Figure S10. The permeability profile of PER dissolved in octyl dodecanol using Skin-PAMPA.

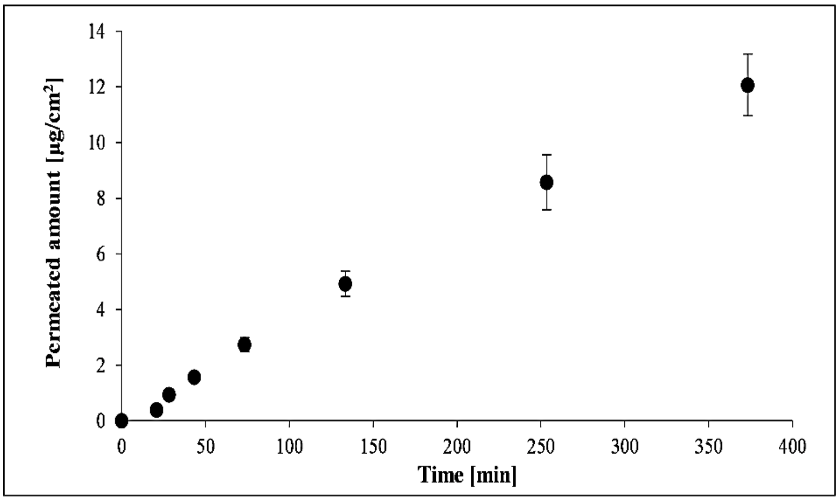

Figure S11. The permeability profile of PER dissolved in apricot kernel oil using Skin-PAMPA.

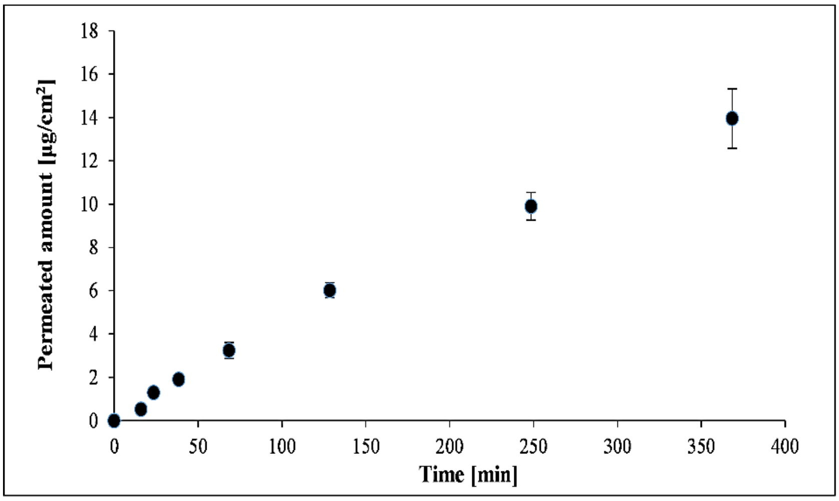

Figure S12. The permeability profile of PER dissolved in corn oil using Skin-PAMPA.
